# Repeat Lumbar Puncture in Adults With Encephalitis: Patient Characteristics, Cerebrospinal Fluid Dynamics, and Diagnostic Yield

**DOI:** 10.1093/ofid/ofag574

**Published:** 2026-09-05

**Authors:** Jordan Benderoth, Ralph Habis, Roba El Zibaoui, Merrick Garner, John C Probasco, Rodrigo Hasbun, Arun Venkatesan

**Affiliations:** Johns Hopkins Encephalitis Center, Department of Neurology, Johns Hopkins University School of Medicine, Baltimore, Maryland, USA; Johns Hopkins Encephalitis Center, Department of Neurology, Johns Hopkins University School of Medicine, Baltimore, Maryland, USA; Johns Hopkins Encephalitis Center, Department of Neurology, Johns Hopkins University School of Medicine, Baltimore, Maryland, USA; Department of Medicine, Section of Infectious Disease, McGovern Medical School, UTHealth Science Center, Houston, Texas, USA; Johns Hopkins Encephalitis Center, Department of Neurology, Johns Hopkins University School of Medicine, Baltimore, Maryland, USA; Department of Medicine, Section of Infectious Disease, McGovern Medical School, UTHealth Science Center, Houston, Texas, USA; Johns Hopkins Encephalitis Center, Department of Neurology, Johns Hopkins University School of Medicine, Baltimore, Maryland, USA

**Keywords:** autoimmune, cerebrospinal fluid, encephalitis, infectious, lumbar puncture

## Abstract

**Background:**

During an evaluation for encephalitis, lumbar puncture (LP) plays a critical role in establishing a diagnosis. Repeat LP may be needed when the diagnosis remains in doubt, the specific etiology of encephalitis has not yet been identified, or to aid management decisions. However, findings on repeat LP have not been well characterized, potentially confounding interpretation. Here, we examine the clinical characteristics of patients with encephalitis who underwent repeat LP, the evolution of cerebrospinal fluid (CSF) parameters, and the indications for repeat LP during acute hospitalization.

**Method:**

This retrospective study analyzes LP and clinical data from 627 adults with all causes of encephalitis admitted between 2002 and 2022 across 2 hospital systems in Maryland and Texas.

**Results:**

Of the 627 patients, 184 (29.3%) underwent repeat LP, with a median interval of 6 days between procedures, and more commonly among those with more severe presentations. Patients were more likely to undergo repeat LP in autoimmune than in infectious encephalitis (42/113 [37.2%] vs 62/275 [22.5%], *P* = .003). In infectious cases, CSF white blood cell counts decreased when repeat LP occurred ≥7 days after initial LP (*P* = .016), whereas no decrease occurred in autoimmune cases. Repeat LP yielded a new diagnosis in 16/184 (8.6%). Among patients without initial pleocytosis, 51.4% developed pleocytosis on repeat LP, associated with worse outcomes. Diagnostic uncertainty was the most common indication.

**Conclusions:**

Repeat LPs are performed in one-third of encephalitis hospitalizations and are more common in patients with autoimmune etiologies, severe disease, or diagnostic uncertainty. Cerebrospinal fluid dynamics differ between infectious and autoimmune etiologies.

## Teaser

We describe characteristics of patients with encephalitis who receive repeat lumbar punctures (LPs) during hospitalization and analyze cerebrospinal fluid profiles over time across encephalitis subtypes. Reasons for performing repeat LPs, along with diagnostic yield, are characterized, including in patients who initially lack pleocytosis.

Encephalitis is a severe neurological condition that requires timely diagnosis and intervention to optimize patient outcomes [1–4]. However, definitive etiological testing often requires several days and may be inconclusive, necessitating diagnostic frameworks that incorporate clinical features, imaging, serological markers, and cerebrospinal fluid (CSF) findings to guide early management [1, 4, 5]. While CSF pleocytosis remains a key indicator of central nervous system (CNS) inflammation, up to one-fourth of patients with encephalitis may present without pleocytosis on their initial lumbar puncture (LP), complicating early identification and potentially delaying empirical management [6]. Given that a single nondiagnostic CSF evaluation may not exclude an underlying infectious or neuroinflammatory process, the Infectious Diseases Society of America (IDSA) guidelines highlight the possibility of early false-negative herpes simplex virus (HSV) polymerase chain reaction (PCR) results, particularly in HSV-1 encephalitis, and recommend repeat testing within 3–7 days when clinical suspicion persists [1, 7, 8]. Similar recommendations have been incorporated into French guidelines, especially when the initial LP is performed within 4 days of symptom onset, when diagnostic sensitivity may be reduced [9]. Although most evidence derives from HSV-1 encephalitis, early false-negative PCR results have also been reported in cases of neuroinvasive HSV-2 [10]. Likewise, a repeat LP may expand diagnostic evaluation, monitor CSF inflammatory changes, and assist in managing complications such as increased intracranial pressure.

In this retrospective study, we aim to examine the use of repeat LP during the acute hospitalization of people with all causes of encephalitis. Our objectives are to describe the clinical characteristics of patients undergoing repeat LP, characterize changes in CSF parameters over time, identify the indications for repeat LP, and determine the role of repeat LP in identifying the etiology of encephalitis. We hypothesized that patients who underwent repeat LP would have distinct clinical characteristics compared with those who did not, reflecting greater disease severity or diagnostic uncertainty, and that changes in CSF parameters over time would differ between patients with infectious encephalitis (IE) and those with autoimmune encephalitis (AE).

## METHODS

### Study Population

People aged 18 years or older with a diagnosis related to encephalitis at discharge were identified from 2 hospital systems in Houston, Texas, spanning from February 2005 to February 2023, and from Johns Hopkins Hospital and Johns Hopkins Bayview Medical Center in Baltimore, Maryland, between 1 June 2006 and 15 March 2016. Patients were ascertained using the diagnosis coding system in use at the time of hospitalization, with the 9th Edition of the International Classification of Diseases Clinical Modification (ICD-9-CM) codes used before October 2015 and ICD-10-CM thereafter. Administrative codes were used solely for initial case ascertainment. To minimize potential misclassification, all identified cases underwent comprehensive manual chart review by study coordinators to remove duplicate records, identify cases potentially missed during the transition between coding systems, and confirm that all included patients met established diagnostic criteria for encephalitis. A complete list of ICD-9-CM and ICD-10-CM codes used for case identification is provided in Supplementary Table 1. Additional patients were prospectively enrolled from Johns Hopkins Hospital and its outpatient center between January 2016 and December 2022.

**Figure 1.**
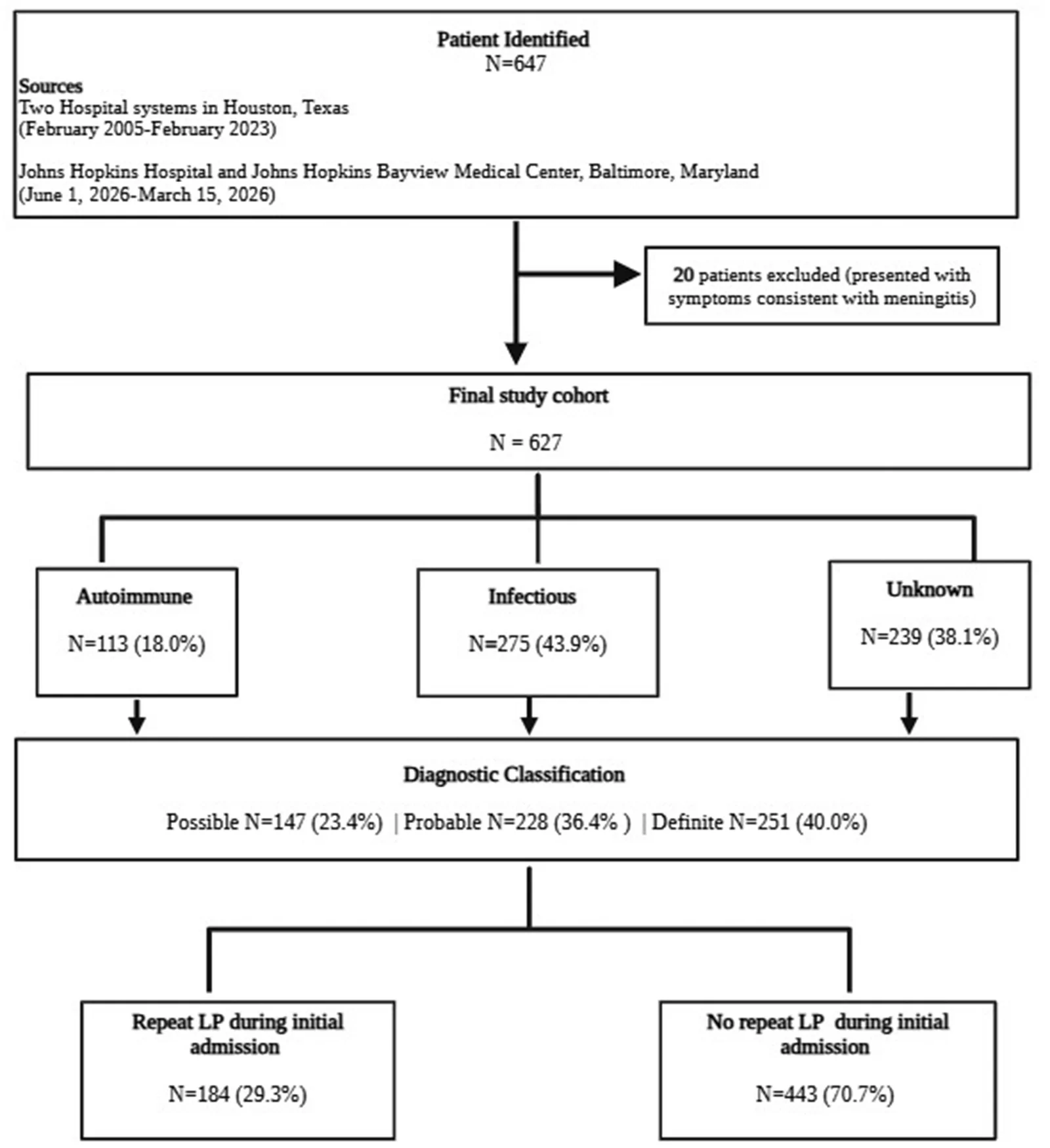
Study cohort flow diagram illustrating patient inclusion, etiologic classification, and repeat lumbar puncture status. Figure created on BioRender.com.

Patients presenting with initial symptoms consistent with meningitis, rather than encephalitis, were excluded. The final cohort included a total of 627 patients who met the International Encephalitis Consortium (IEC) criteria and/or Graus et al criteria for encephalitis (Figure 1) [1, 4]. This study represents a secondary analysis of a previously published cohort with an updated case identification strategy incorporating ICD-10 codes to capture additional eligible cases and address a distinct research question. Institutional Review Board approval was obtained from the University of Texas and Johns Hopkins Hospital [6].

### Data Extraction and Definition of Variables

Data was extracted as previously described [6]. Patients transferred from outside institutions were excluded if information regarding their initial diagnostic evaluation or CSF results was unavailable. Clinical, laboratory, diagnostic, and outcome data were collected from the index hospitalization. Symptom onset was categorized as acute (≤5 days from symptom onset to presentation) or insidious (>5 days).

Cerebrospinal fluid characteristics were collected from all LPs performed during hospitalization, including white blood cell (WBC) count and differential, red blood cell (RBC) count, glucose, and protein. Pleocytosis was defined as a CSF WBC count ≥ 5 cells/mm^3^. Diagnostic testing performed on CSF was also collected, including standalone viral PCR assays, arboviral IgM testing, and AE antibody panels when available.

For patients undergoing repeat LP, the documented clinical rationale was extracted from provider notes and categorized into 5 predefined groups: diagnostic uncertainty, clinical deterioration or lack of response, intracranial pressure (ICP) management, monitoring treatment response, and other. Multiple indications could be assigned to a single repeat LP. Diagnostic uncertainty was defined as a repeat CSF evaluation performed when the underlying etiology remained unresolved, including consideration of early false-negative HSV PCR results or initially nondiagnostic CSF findings [6, 9].

The timing of repeat LP was evaluated relative to the initial LP and categorized as occurring within 0–7 days or >7 days. Although no universal guideline specifies the optimal interval for CSF reassessment across encephalitis etiologies, this cutoff was chosen to capture the early diagnostic reassessment period, in accordance with recommendations to repeat CSF analysis, including HSV PCR testing, within 3–7 days when clinical suspicion remains high despite an initially negative or nondiagnostic evaluation [1, 11, 12].

Additional clinical variables collected were the Charlson Comorbidity Index (CCI), Glasgow Coma Scale (GCS) at admission, and immunosuppression status. The CCI was derived from documented medical history, with scores ≥ 3 considered indicative of a high comorbidity burden. The level of consciousness was assessed using the documented GCS at admission or estimated from available neurological examination findings, when not explicitly mentioned. Immunosuppression was defined as Human Immunodeficiency Virus (HIV) infection irrespective of Cluster of Differentiation 4 (CD4) count, recent chemotherapy, solid-organ or hematopoietic stem cell transplantation, congenital immunodeficiency, or use of immunosuppressive or immunomodulatory therapy for >1 month before encephalitis onset, including prednisone ≥ 20 mg/day (or equivalent) or agents such as mycophenolate mofetil, tacrolimus, methotrexate, sulfasalazine, adalimumab, or rituximab [13]. Clinical variables, including presenting symptoms, seizures, intensive care unit (ICU) admission, and encephalitis etiology, were also extracted from medical records during the initial hospitalization. Finally, outcomes recorded included hospital length of stay, mortality, and functional status at discharge, which was assessed using the Glasgow Outcome Scale (GOS), a validated 5-point ordinal scale ranging from 1 (death) to 5 (good recovery), with higher scores indicating better functional outcomes [14].

### Statistical Analysis

Univariate analyses were performed to compare CSF profile parameters between the first lumbar puncture (LP1) and subsequent follow-up LPs among patients who underwent repeat LP. Temporal changes in CSF findings were assessed according to the interval between LPs, with follow-up results categorized based on the time elapsed from the initial LP. Differences between LP1 and follow-up CSF values were evaluated using the Wilcoxon signed-rank test. Additionally, clinical characteristics were compared between patients who underwent repeat LP and those who did not. A separate analysis was performed among patients without initial pleocytosis to compare those who developed pleocytosis on follow-up LP with those who did not. Categorical variables were analyzed using the χ² test or Fisher's exact test, as appropriate, while continuous variables were evaluated using the Mann–Whitney U test. The dataset was assessed for completeness and contained minimal missing data; missing variables were verified through available record review when possible, and remaining missing values were handled using pairwise deletion to maximize available observations while minimizing potential loss of information and selection bias. All analyses were conducted using Statistical Package for the Social Sciences (SPSS) version 29, with statistical significance defined as 2-sided *P* < .05.

## RESULTS

### Clinical Characteristics of Patients Undergoing Repeat Lumbar Puncture

**Figure 2.**
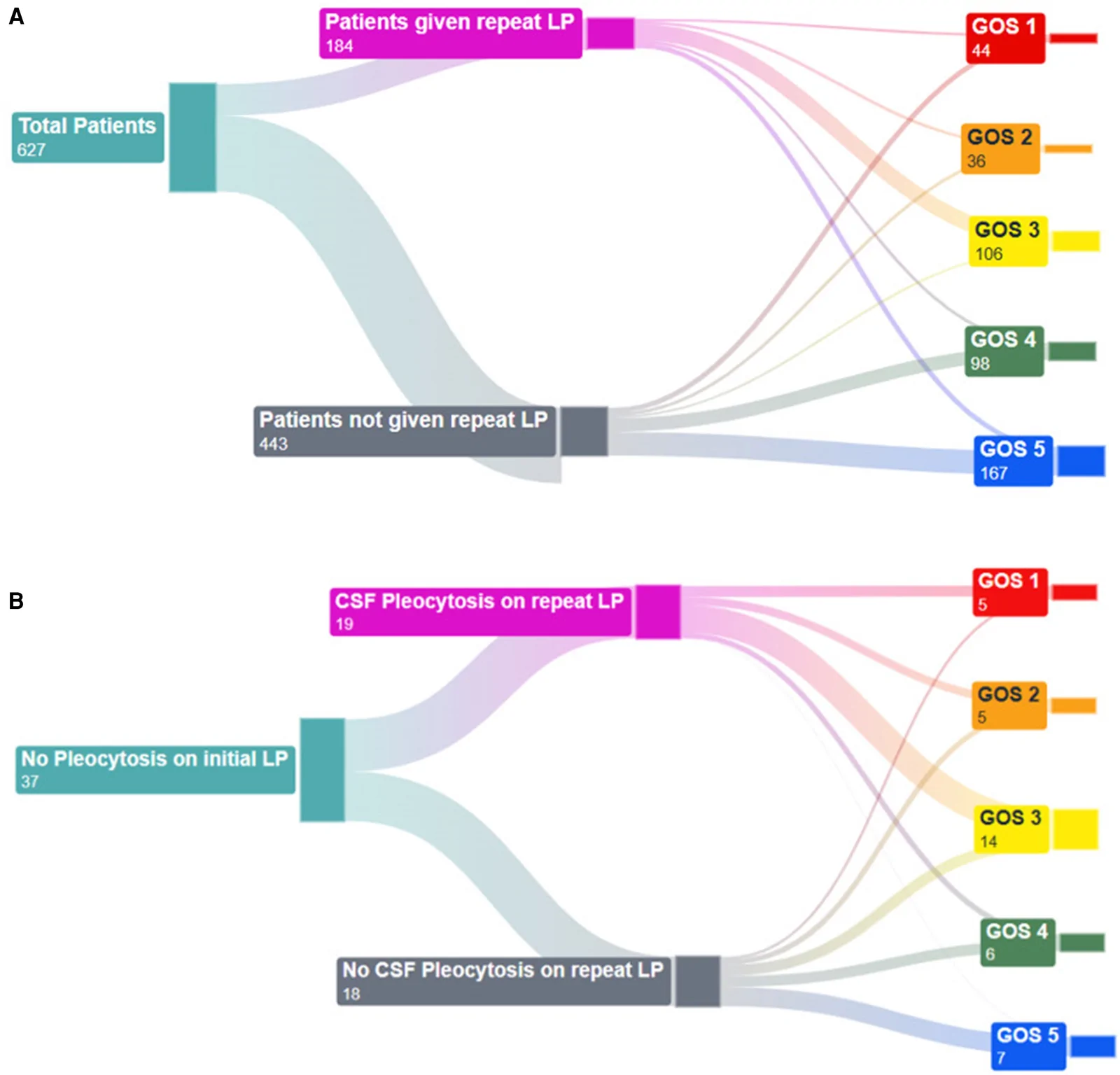
Sankey plot illustrating clinical outcome stratified by (*A*) patients with vs without repeat lumbar puncture and (*B*) by the presence or absence of CSF pleocytosis on initial LP. Figure created with sankeyMATIC.com. Abbreviations: CSF, cerebrospinal fluid; LP, lumbar puncture.

Of the 627 patients included in this cohort, 184 (29.3%) received a repeat LP during their initial admission (Table 1). The median time to LP2 was 6 days [IQR: 3–10 days]. The distribution of encephalitis subtypes differed between groups, with patients undergoing repeat LP demonstrating a higher proportion of AE (22.8% vs 16.0%, *P* = .052) and a lower proportion of IE (33.7% vs 48.1%, *P* < .001). The proportion of cases with unknown etiology was similar between groups (43.5% vs 35.9%, *P* = .086). Patients undergoing repeat LP were also more likely to have insidious onset of symptoms compared to those with a single LP (44.9% vs 53.9%, *P* = .044; see METHODS for definition). Furthermore, those with follow-up LP had a higher incidence of seizures (46.9% vs 38.1%, *P* = .047), refractory status epilepticus (5.8% vs 0.7%, *P* < .001), and abnormal electroencephalography (EEG) findings (83.6% vs 74.4%, *P* = .030). Finally, those with repeat LP were also more frequently admitted to the ICU (55.2% vs 34.5%, *P* < .001) and had longer length of stay (23 vs 9 days, *P* < .001) and lower GOS at discharge (Table 2; Figure 2*A*).

**Table 1.** Characteristics of Patients Who Received a Repeat Lumbar Puncture Compared With Those Who Did Not Receive a Repeat Lumbar Puncture

	All Patients (N = 627)	Patients Not Given Repeat LP (N = 443)	Patients Given Repeat LP (N = 184)	*P* Value^a^
Demographics				
Gender				
Male	311/627 (49.6)	212/443 (47.9)	99/184(53.8)	.160
Female	316/627 (50.4)	231/443 (52.1)	85/184 (46.5)	
Age^b^ median [IQR], y	49 [33.0–63.0]	49 [32.0–63.0]	50 [33.0–62.8]	.967
Diagnostic criteria				
Possible	147/627(23.4)	117/443(26.4)	30/184(16.3)	.007*
Probable	228/627 (36.4)	156/443(35.2)	72/184 (39.1)	.363
Definite	251/627 (40.0)	170/443 (38.4)	81/184 (44.0)	.210
Subtype encephalitis				
Autoimmune	113/627 (18.0)	71/443 (16.0)	42/184 (22.8)	.052*
Infectious	275/627 (43.9)	213/443 (48.1)	62/184 (33.7)	<.001*
Unknown	239/627 (38.1)	159/443 (35.9)	80/184 (43.5)	.086
Immunocompromised^c^	142/627 (38.1)	94/443 (21.2)	48/184 (26.1)	.190
CCI, median [IQR]	1 [0.0–4.0]	1.[0.0–3.0]	1 [0.0–4.0]	.703
CCI ≥ 3	215/627 (34.4)	151/443 (34.1)	64/184 (34.7)	.433
Clinical presentation				
Acute onset	306/597 (51.3)	226/419 (53.9)	80/178 (44.9)	.044*
Presence of fever	325/597 (54.4)	229/423 (54.1)	96/174 (55.2)	.857
Seizures	248/610 (40.6)	164/431 (38.1)	84/179 (46.9)	.047*
Status epilepticus	57/620 (9.2)	30/440 (6.8)	27/180 (15.0)	.001*
Refractory status epilepticus	13/609 (2.1)	3/436 (0.7)	10/173 (5.8)	<.001*
Headache	288/547 (52.7)	205/386 (53.1)	83/161 (51.6)	.778
Altered mental status	479/620 (77.3)	317/438 (72.4)	162/182 (89.0)	<.001*
Focal neurological signs^d^	239/619 (38.6)	164/437 (37.5)	75/182 (41.2)	.415
Psych symptoms on admission	304/622 (48.9)	206/439 (46.9)	98/183 (53.6)	.136
Memory deficits	188/616 (30.5)	126/436 (28.9)	62/180 (34.4)	.170
GCS, median [IQR]	14 [11.0–15.0]	14 [12.0–15.0]	14 [11.0–15.0]	.480
Imaging and EEG findings				
Cerebral edema	74/622 (11.9)	53/441 (12.0)	21/181 (11.6)	.880
Abnormal MRI	309/528 (58.5)	206/361 (57.1)	103/167 (61.7)	.320
Abnormal EEG	339/437 (77.6)	212/285 (74.4)	127/152 (83.6)	.030*
Epileptiform activity on EEG	96/404 (23.8)	63/266 (23.7)	33/138 (23.9)	.900
Laboratory findings				
Leukocytosis (serum WBC count > 11 × 10^3^/μL)	198/601 (32.9)	131/421 (31.1)	67/180 (37.2)	.150
Opening pressure on LP1, median [IQR], mmH2O	20 [15.0–27.0]	20 [15.1–27.8]	19 [14–26.3]	.279
Abnormal CSF cutoffs on LP1				
WBC > 5 (cells/mm^3^)	458/627 (73.0)	314/443 (70.9)	144/184 (78.3)	.058
Protein > 45 (mg/dL)	463/619 (74.8)	325/439 (74.0)	138/180 (76.7)	.490
Glucose < 50 (mg/dL)	148/606 (24.4)	100/429 (23.3)	48/177 (27.1)	.320
CSF median data (LP1)				
WBC, median [IQR], (cells/mm^3^)	27 [4.0–163.0]	25 [4.0–169.0]	38 [7.3–144.3]	.256
Neutrophil, median [IQR], (%)	3 [0.0–36.0]	3 [0.0–39.3]	3 [0.0–31.5]	.526
Lymphocyte, median [IQR], (%)	79 [29.0–93.0]	78 [27.8–93.0]	79 [30.0–94.0]	.784

Data are presented as n/N (%) unless otherwise indicated. “*” indicates statistical significance or *P* < .05.

Abbreviations: CCI, Charlson Comorbidity Index; CSF, cerebrospinal fluid; EEG, electroencephalography; GCS, Glasgow Coma Scale; GOS, Glasgow Outcome Scale; ICU, intensive care unit; IQR, interquartile range; LOS, length of stay; LP, lumbar puncture (LP1, first lumbar puncture during acute admission); MRI, magnetic resonance imaging; WBC, white blood cell.

^a^ *P* value comparing patients not given repeat LP with those given repeat LP.

^b^Age range, 18–92 years.

^c^Immunocompromised is defined as HIV, recent chemotherapy ( < 1 m), solid-organ or bone marrow transplantation, receiving ≥20 mg of prednisone or equivalent for >1 m, or congenital immunodeficiency.

^d^Self-reported symptoms suggestive of focal neurological manifestations, such as acute-onset cranial nerve abnormalities or acute defects in sensorimotor abilities, including aphasia.

**Table 2.** Hospitalization Outcomes of Patients Who Received a Repeat Lumbar Puncture Compared With Those Who Did Not Receive a Repeat Lumbar Puncture

	All Patients (N = 627)	Patients Not Given Repeat LP (N = 443)	Patients Given Repeat LP (N = 184)	*P* Value^a^
Outcomes				
LOS, median [IQR], d	12 [6.0–23.0]	9 [5.0–16.0]	23 [7.0–40.5]	< .001*
ICU admission	251/618 (40.6)	150/435 (34.5)	101/183 (55.2)	< .001*
Glasgow Outcome Scale				
1—death	44/565 (7.8)	30/393 (7.6)	14/172 (8.1)	.607
2—persistent vegetative state	36/565 (6.4)	19/393 (4.8)	17/172 (9.9)	.022*
3—severe disability	221/565 (39.1)	127/393 (32.3)	94/172 (54.7)	< .001*
4—moderate disability	98/565 (17.3)	80/393 (20.4)	18/172 (10.5)	.011*
5—good recovery	167/565 (29.6)	138/393 (35.1)	29/172 (16.9)	< .001*

Data are presented as n/N (%) unless otherwise indicated. “*” indicates statistical significance or *P* < .05.

Abbreviations: ICU, intensive care unit; IQR, interquartile range; LOS, length of stay; LP, lumbar puncture.

^a^ *P* value comparing patients not given repeat LP with those given repeat LP.

### Reason for Follow-Up Lumbar Punctures

The indication for a repeat LP was available for 175/184 (95.1%) of patients. Diagnostic uncertainty was the most common indication, frequently occurring in combination with clinical deterioration or lack of response to treatment (Table 3). The combination of clinical deterioration/lack of response and ICP monitoring was observed more frequently in IE (6 cases) compared with AE (2 cases) and encephalitis of unknown etiology (1 case).

**Table 3.** Reason for Follow-Up Lumbar Puncture Over Time

	Reason^a^	Frequency	Percent (%)
**0–7** d**ays from LP1**	Diagnostic uncertainty	89/123	72.4
	Clinical deterioration or lack of response	31/123	25.2
	Monitoring treatment response	17/123	13.8
	ICP monitoring	7/123	5.7
	Other	6/123	4.9
**>7** d**ays from LP1**	Diagnostic uncertainty	86/139	61.9
	Clinical deterioration or lack of response	44/139	31.7
	Monitoring treatment response	45/139	32.4
	ICP monitoring	5/139	3.6
	Other	1/139	0.7
**Overall**	Diagnostic uncertainty	175/262	66.8
	Clinical deterioration or lack of response	75/262	28.6
	Monitoring treatment response	62/262	23.7
	ICP monitoring	12/262	4.6
	Other	7/262	2.7

Abbreviations: ICP, intracranial pressure; LP, lumbar puncture (LP1, first lumbar puncture during acute admission).

^a^Reason for follow-up lumbar puncture over time, comparing reasons for follow-up lumbar puncture taken within 1 week of admission lumbar puncture and those with follow-up lumbar puncture taken after 1 week of admission lumbar puncture.

### Evolution of Cerebrospinal Fluid Parameters

**Figure 3.**
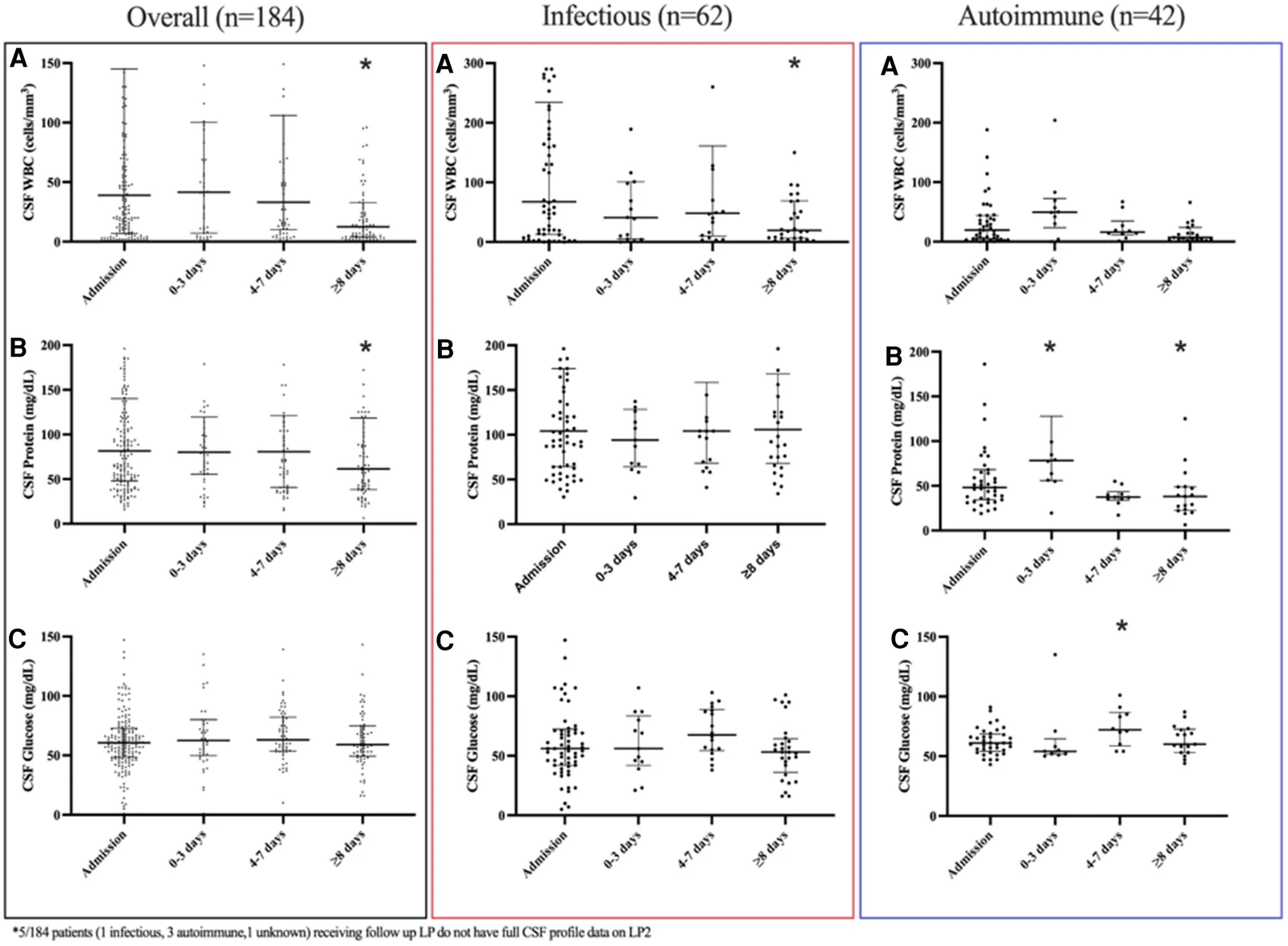
CSF profiles of encephalitis patients over time. “*” indicates the first time point with significantly (*P* < .05) normalized inflammatory markers. *A–C*, CSF profile results of patients' admission lumbar puncture (LP1) compared to follow-up LP (LP2) with Mann–Whitney U statistical analysis. (*A*), CSF WBC (cells/mm^3^) over time, (*B*) CSF protein (mg/dL) over time, (*C*) CSF glucose (mg/dL) over time. Abbreviations: CSF, cerebrospinal fluid; WBC, white blood cell.

Among the 184 patients who underwent repeat LP, we characterized changes in CSF parameters over time (Table 4). Overall, there was a decrease in median WBC counts on repeat LP (LP1 WBC = 38.0 [IQR: 7.3–144.3], LP2 WBC = 19.0 [IQR: 5.5–67.0], *P* < .001). Protein levels also showed a decrease on repeat LP (LP1 protein = 80.0 [IQR: 48–137], LP2 protein = 75.0 [IQR: 41.0–120.0], *P* = .002). However, RBC and glucose levels did not change. On stratification by encephalitis subtype, infectious cases showed a significant decrease in CSF WBC count between LP1 and LP2, whereas no significant change was observed in autoimmune cases (67.5 [12.8–234.3] vs 39.0 [7.0–88.0]; *P* = .002 vs *P* = .199). To better understand the timing of these changes in CSF parameters, we categorized the LP2 results into predefined time intervals based on the number of days elapsed since LP1 (Figure 3). In the overall cohort, both WBC and protein decreased on repeat LP when more than 7 days had passed since the initial LP (*P* < .001 for WBC and *P* = .037 for protein). Cerebrospinal fluid WBC counts decreased significantly in the infectious group when LP2 was performed >7 days after LP1 (*P* = .016), whereas no significant change was observed in the autoimmune group. In contrast, autoimmune cases demonstrated a trend toward increased WBC counts when LP2 was performed within 3 days of LP1.

**Table 4.** Comparison of CSF Profiles on First Lumbar Puncture to Follow-Up Lumbar Puncture

	LP1 (Given Pt. Had LP2)	LP2	*P* Value^a^
WBC median (cells/mm^3^)	38.0 [7.3–144.3]	19.0 [5.5–67.0]	<.001*
Infectious	67.5 [12.8–234.3]	39.0 [7.0–88.0]	.002*
Autoimmune	20.0 [5.0–44.0]	14.0 [5.0–42.0]	.199
Neutrophil median (%)	3.0 [0.0–31.5]	1.0[0.0–9.3]	.028*
Infectious	7.0 [0.0–42.0]	2.0 [0.5–7.0]	.010*
Autoimmune	0.0 [0.0–3.0]	0.0 [0.0–2.0]	.410
Lymphocyte median (%)	79 [30.0–94.0]	89.5[67.0–96.0]	<.001*
Infectious	61.0 [25.5–86.3]	87.0 [59.0–95.3]	<.001*
Autoimmune	91.0 [64.3–95.8]	93.0 [83.0–98.0]	.157
RBC (cells/mm^3^)	18.0 [3.0–189.0]	20.0 [3.0–253.5]	.762
Infectious	20.0 [4.5–462.0]	22.0 [5.0–184.3]	.636
Autoimmune	15.0 [1.8–61.0]	27.0 [3.5–651.8]	.096
Protein (mg/dL)	80.0 [48.0–137.0]	75.0 [41.0–120.0]	.002*
Infectious	104.1 [64.1–173.9]	104.0 [67.0–142.8]	.104
Autoimmune	48.0 [34.4–68.0]	40.0 [29.4–64.3]	.308
Glucose (mg/dL)	61.0[49.0–73.0]	60.0 [52.0–80.0]	.151
Infectious	56.0 [42.0–72.3]	56.0 [45.0–85.0]	.269
Autoimmune	61.0 [54.0–68.5]	60.0 [53.3–73.0]	.271

Data are presented as median [IQR]. “*” indicates statistical significance or *P* < .05.

Abbreviations: LP, lumbar puncture (LP1, first lumbar puncture during acute admission; LP2, first follow-up lumbar puncture during acute admission); RBC, red blood cell; WBC, white blood cell.

^a^ *P* value comparing CSF profiles from patients' admission lumbar punctures with those from the same patients' follow-up lumbar punctures.

A subgroup analysis was also done to determine the association between empiric steroid therapy and changes in CSF WBC count on repeat LP. Among the 184 patients with repeat LP, 127 (69.0%) received corticosteroids during hospitalization, including 39 (21.2%) treated empirically before LP2. The median time from steroid treatment to LP2 was 6 days [IQR: 2.3–11.3 days]. Patients who received empiric steroids were younger, with a median age of 40.5 years vs 49.0 years (*P* = .009) and had fewer seizures on presentation (33.3% vs 59.3%, *P* = .012). On LP2, an increase in CSF WBC count was observed in 27.0% (10/37) of patients who received corticosteroids before repeat LP compared with 45.9% (39/85) of those who did not. In contrast, CSF WBC count decreased or remained unchanged in 73.0% (27/37) and 54.1% (47/85), respectively. Nevertheless, neither difference was statistically significant (*P* = .070). Additional demographic and clinical characteristics are provided in Supplementary Table 2.

### Role of Follow-Up Testing in Identification of Etiology

Sixteen out of 184 (8.6%) patients had a new diagnosis defined in the CSF on follow-up LP. Of these, 10 (62.5%) had infectious causes, while 6 had autoimmune causes. Among the cases of IE, 4 had initially negative CSF evaluations, with a pathogen subsequently identified on repeat LP. These included 2 patients with West Nile virus (WNV) and 1 patient each with HSV and varicella-zoster virus (VZV) infection, respectively (Table 5).

**Table 5.** Patients With New Diagnosis From Follow-Up CSF Testing

Infectious Cases (n = 10)	Autoimmune Cases (n = 6)
West Nile virus (WNV) (4)^a^	NMDA (5)
Herpes simplex virus (HSV) (2)^a^	Anti-GAD65 (1)
Varicella-zoster virus (VZV) (2)^a^	…
Syphilis (1)	…
Human herpesvirus 6 (HHV6) (1)	…

Abbreviations: NMDA, N-methyl-D-aspartate; GAD65, Glutamic Acid Decarboxylase 65.

^a^2/4 WNV, 1/2 HSV, and 1/2 VZV cases had negative testing on previous lumbar puncture (LP).

### Follow-Up Lumbar Puncture in Herpes Simplex Virus Encephalitis

We next examined the subgroup of patients with HSV encephalitis who underwent a follow-up LP. Seventeen patients with HSV encephalitis underwent repeat CSF analysis (median 12 days [IQR: 7–20] from initiation of acyclovir). Half of these patients (9/17, 52.9%) exhibited a decrease in CSF WBC count, with 2 demonstrating normocellular CSF upon repeat LP. Of the 16 patients with HSV PCR testing on LP2, 9 had a positive PCR and 7 had a negative PCR; no association was found between PCR positivity and the interval between initiation of acyclovir and LP2.

### Follow-Up Lumbar Puncture Testing in Patients Without Pleocytosis on Initial Lumbar Puncture

Among the 169/627 (26.9%) patients without pleocytosis (CSF WBC ≥ 5 cells/µL) on LP1, 37/169 (21.9%) underwent repeat LP. Of these, 19/37 (51.3%) developed pleocytosis on repeat LP. Notably, patients with AE accounted for 6/19 (31.6%) of these cases, despite representing only 18% of the overall cohort. Compared with patients who remained without pleocytosis, those who developed pleocytosis on repeat LP had a longer median hospital length of stay of 51 days [IQR: 29.0–76.0] vs 17 days [IQR: 11.3–34.0], *P* = .005. Similarly, good recovery at discharge (GOS score 5) was observed in 7/18 (38.9%) patients who remained without pleocytosis, compared with 0/19 (0%) patients who developed pleocytosis on LP2 (*P* = .020) (Table 6; Figure 2*B*).

**Table 6.** Characteristics of Patients Who Developed Pleocytosis on Follow-Up Lumbar Puncture vs Those Who Never Developed Pleocytosis

	Patients Who	Patients Who	*P* Value^a^
	Developed Pleocytosis	Never Developed	
	(n = 19)	Pleocytosis (n = 18)	
Demographics			
Gender			
Male	10/19 (52.6)	10/18 (55.6)	.858
Female	9/19 (47.4)	8/18 (44.4)	
Age^b^ median [IQR], y	59[23.0–67.0]	50.5 [40.8–63.8]	.663
Subtype encephalitis			
Autoimmune	6/19 (31.6)	5/18 (27.8)	.800
Infectious	7/19 (36.8)	4/18 (22.2)	.331
Unknown	6/19 (31.6)	9/18 (50.0)	.254
Immunocompromised^d^	6/19 (31.6)	5/18 (27.8)	.800
CCI, median [IQR]	3.0 [0.0–5.0]	2.0 [0.0–5.0]	.778
CCI ≥ 3	10/19 (52.6%)	7/18 (38.9%)	.525
Clinical presentation			
Acute onset	7/18 (38.9)	4/15 (26.7)	.458
Presence of fever	7/19 (36.8)	4/17 (23.5)	.387
Seizures	11/19 (57.9)	10/17 (58.8)	.955
Status epilepticus	4/18 (22.2)	3/17 (17.6)	.735
Refractory status epilepticus	3/18 (16.7)	1/17 (5.9)	.316
Headache	7/17 (41.2)	5/16 (31.3)	.554
Altered mental status	18/19 (94.7)	14/17 (82.4)	.238
Focal neurological signs^e^	11/19 (57.9)	10/17 (58.8)	.955
Psych symptoms on admission	13/19 (68.4)	14/18 (77.8)	.522
Memory deficits	9/19 (47.4)	8/17 (47.1)	.985
Cerebral edema	1/19 (5.3)	1/17 (5.9)	.935
GCS, median [IQR]	14 [11.5–15.0]	14 [12.0–15.0]	.623
Outcomes			
LOS^c^, median [IQR]	51 [29.0–76.0]	17[11.3–34.0]	.005*
ICU admission	14/19 (73.7)	10/18 (55.6)	.248
Glasgow Outcome Scale			
1—death	4/19 (21.1)	1/18 (5.6)	.340
2—persistent vegetative state	3/19 (15.8)	2/18 (11.1)	.527
3—severe disability	10/19 (52.6)	4/18 (22.2)	.058
4—moderate disability	2/19 (10.5)	4/18 (22.2)	.405
5—good recovery	0/19 (0.0)	7/18 (38.9)	.020*

Data are presented as n/N (%) unless otherwise indicated. “*” indicates statistical significance or *P* < .05.

Abbreviations: CCI, Charlson Comorbidity Index; CSF, cerebrospinal fluid; EEG, electroencephalography; GCS, Glasgow Coma Scale; GOS, Glasgow Outcome Scale; ICU, intensive care unit; IQR, interquartile range; LOS, length of stay; LP, lumbar puncture (LP1, first lumbar puncture during acute admission); MRI, magnetic resonance imaging; WBC, white blood cell.

^a^ *P* value comparing the patients who developed pleocytosis with patients who never developed pleocytosis.

^b^Age range, 18–92 y.

^c^Length of stay at the hospital.

^d^Immunocompromised is defined as HIV, recent chemotherapy (<1 m), solid-organ or bone marrow transplantation, receiving ≥20 mg of prednisone or equivalent for >1 m, or congenital immunodeficiency.

^e^Self-reported symptoms suggestive of focal neurological manifestations, such as acute-onset cranial nerve abnormalities or acute defects in sensorimotor abilities, including aphasia.

## DISCUSSION

In this multicenter cohort, we evaluated the frequency, indications, diagnostic yield, and longitudinal CSF changes associated with repeat LP in hospitalized people with encephalitis. Overall, 30% of patients underwent repeat LP during the initial hospitalization, most commonly due to diagnostic uncertainty. Patients undergoing repeat LP had higher rates of seizures, refractory status epilepticus, abnormal electroencephalographic findings, ICU admission, longer hospital stays, and worse discharge outcomes. A repeat LP was more frequently performed in people with AE than IE. Despite repeat LP, 90% of CSF-based encephalitis diagnoses were established from the initial LP, with additional diagnostic information identified in a minority of cases. Serial CSF analysis demonstrated differing patterns of change between IE and AE, with declining CSF WBC counts in IE and persistent CSF inflammation in AE. Among patients without initial pleocytosis, more than half developed pleocytosis on repeat LP.

These findings expand the limited literature regarding repeat LP in encephalitis. Most previous studies have focused on bacterial meningitis, where repeat LP is performed in only approximately 8% of patients and is typically prompted by clinical deterioration, persistent infection, or management of complications, such as hydrocephalus, rather than diagnostic uncertainty [11]. In contrast, encephalitis frequently presents nonspecifically, leaving many patients without a definitive etiology after the initial evaluation, which explains the higher frequency of repeat LP observed in encephalitis and reinforces the need to develop evidence-based approaches to guide its use.

The greater use of repeat LP among people with AE is consistent with prior studies, demonstrating that CSF findings in this heterogeneous and expanding group of antibody-mediated disorders may be atypical, evolve, and show less consistent inflammatory abnormalities than those observed in IE [5, 15, 16]. A previous study of patients with neuronal surface antibody-associated encephalitis reported pleocytosis in only 36.6% of patients at presentation, and approximately 50% of follow-up LPs demonstrated increasing pleocytosis [17]. Our findings build on previous reports demonstrating that CSF inflammatory abnormalities are absent in a substantial proportion of people with AE at presentation and may emerge only on follow-up analysis, likely reflecting the evolving nature of neuroinflammation and potential delays in initiating appropriate immunosuppressive therapy. However, despite growing recognition of this evolving inflammatory profile, current best-practice recommendations provide little guidance regarding the role or timing of repeat LP in suspected AE [1, 12, 16, 18, 19].

Likewise, a repeat LP is also valuable in selected cases of IE. In our study, among the 16 patients in whom repeat LP established a new diagnosis, 1 patient (6.25%) with HSV encephalitis had an initially negative HSV PCR result that became positive on repeat CSF testing. These findings are consistent with a recent multicenter study reporting a 4% biological false-negative rate for CSF HSV PCR, with false-negative results occurring most commonly after early sampling within 4 days of symptom onset, although late testing, low CSF viral load, imperfect assay sensitivity, and HSV neutralization by antibodies have also been implicated [9, 20, 21]. Because the interval from symptom onset to the initial LP was not consistently available in our retrospective cohort, we could not determine whether a similar mechanism accounted for these cases. Nevertheless, our findings support current recommendations to repeat HSV PCR 3–7 days after an initial negative result when clinical suspicion for HSV encephalitis remains high, particularly in patients with compatible clinical features or temporal lobe abnormalities on neuroimaging. In addition, given the typical 14- to 21-day duration of antiviral therapy, a repeat LP at the end of treatment may provide an opportunity to evaluate treatment response in selected patients [1, 18].

Nevertheless, evolving pleocytosis on repeat LP should be interpreted cautiously, as recent data demonstrate that up to one-third of patients with non-inflammatory neurological disorders developed mild pleocytosis following repeat LP performed within 10 days of the initial procedure, accompanied by modest increases in CSF protein and the CSF-to-serum albumin quotient, suggesting transient disruption of the blood–CSF barrier [22]. Given that the median interval between LPs in our cohort was 6 days, procedure-related inflammatory changes may have contributed to some cases of newly detected pleocytosis. However, among patients with confirmed encephalitis, evolving neuroinflammation remains the most likely explanation for these longitudinal CSF changes.

Collectively, these findings have several important clinical implications. The high diagnostic yield of the initial LP emphasizes the importance of comprehensive CSF evaluation at presentation, including early autoimmune testing when clinically indicated. Given that 90% of CSF-based diagnoses were established from the initial LP, a repeat LP done solely to increase diagnostic yield is unlikely to provide substantial benefit for most patients. Instead, repeat LP may be most informative in selected scenarios, including persistent suspicion for HSV encephalitis despite an initially negative PCR, evolving clinical or CSF features suggestive of AE, or evaluation for alternative diagnoses such as CNS malignancy [23]. Future prospective studies are needed to better define optimal timing for repeat LP in patients with encephalitis.

### Strengths and Limitations

This study represents one of the largest cohorts to date examining repeat LP use and CSF marker progression in patients with encephalitis. By capturing data from multiple institutions over an extended period, our analysis provides a comprehensive overview of the indications, timing, and diagnostic yield of repeat LPs across infectious, autoimmune, and unknown encephalitis etiologies. The sizable sample allowed for subgroup analyses and helped identify differences in infectious compared to autoimmune presentations. Importantly, by assessing CSF changes over defined time intervals, we offer insight into CSF dynamics in the days following the initial LP, which may help guide clinicians in the timing and necessity of repeat procedures.

As with many cohorts of people with all-cause encephalitis, a substantial proportion of our population did not have a confirmed etiology despite extensive diagnostic evaluation. This likely contributed to the underdiagnosis of both IE and AE and limits our ability to draw conclusions about certain subtypes. Additionally, our study is subject to limitations that are typical of retrospective and observational designs. Missing or incomplete CSF data in patient charts, while minor, may have limited the depth and precision of some of our subgroup analyses.

## CONCLUSIONS

Repeat LP is a common component of encephalitis evaluation, performed in approximately one-third of hospitalized patients and most frequently prompted by persistent diagnostic uncertainty, which accounted for two-thirds of documented indications. Despite its frequent use, repeat CSF testing had limited incremental diagnostic yield, with fewer than 10% of patients receiving a new infectious or autoimmune diagnosis from subsequent CSF analysis. Nevertheless, repeat LP provided additional clinical information in selected patients with evolving CSF inflammatory findings, persistent diagnostic suspicion, or unresolved diagnostic questions after the initial evaluation. Although its role in monitoring treatment response remains uncertain, these findings support a selective, clinically guided approach to repeat LP that prioritizes comprehensive initial CSF assessment and targeted reassessment when additional CSF information is likely to influence care.

## Supplementary Material

ofag574_Supplementary_Data

## Supplementary Data

Supplementary materials are available at *Open Forum Infectious Diseases* online. Consisting of data provided by the authors to benefit the reader, the posted materials are not copyedited and are the sole responsibility of the authors, so questions or comments should be addressed to the corresponding author.
