# Supplementary material for: Repeat Lumbar Puncture in Adults With Encephalitis: Patient Characteristics, Cerebrospinal Fluid Dynamics, and Diagnostic Yield
Source: Open Forum Infect Dis. 2026 Sep 5;13(9):ofag574. doi: 10.1093/ofid/ofag574 (PMC13579470; doi:10.1093/ofid/ofag574)
Supplement: Supplementary file 1 [file ofag574_supplementary_data.zip › Supplementary Table 1.docx]

**Supplementary Table 1:**

| **Diagnosis** | **ICD 10** | **ICD 9** |
| --- | --- | --- |
| Tuberculous meningoencephalitis | A17.82 | 013.6 |
| Listerial meningitis and meningoencephalitis | A32.1 | 027.0 |
| Listerial meningoencephalitis | A32.12 | 027.0 |
| Listerial encephalitis, myelitis and encephalomyelitis | A32.12 | 027.0 |
| Meningococcal encephalitis | A39.81 | 036.1 |
| Meningococcal encephalitis, myelitis and encephalomyelitis | A39.81 | 036.1 |
| Actinomycotic encephalitis | A42.82 | 039.8 |
| Late congenital syphilitic encephalitis | A50.42 | 090.41/090.49 |
| Late syphilitic encephalitis | A52.14 | 094.81 |
| Acute paralytic poliomyelitis, unspecified | A80.30 | 045.0 |
| Creutzfeldt-Jakob disease, unspecified; other Creutzfeldt-Jakob disease | A81.00 A81.09 | 046.10/046.19 |
| Subacute sclerosing panencephalitis | A81.1 | 046.2 |
| Rabies, unspecified | A82.9 | 071 |
| Mosquito-borne viral encephalitis | A83 | 062–064 |
| Japanese encephalitis | A83.0 | 063.0 |
| Western equine encephalitis | A83.1 | 062.1 |
| Eastern equine encephalitis | A83.2 | 062.2 |
| St. Louis encephalitis | A83.3 | 062.3 |
| Australian encephalitis | A83.4 | 062.4 |
| California encephalitis | A83.5 | 062.5 |
| Other mosquito-borne viral encephalitis | A83.8 | 062.8 |
| Mosquito-borne viral encephalitis, unspecified | A83.9 | 062.9 |
| Tick-borne viral encephalitis | A84 | 063 |
| Far Eastern tick-borne encephalitis [Russian spring-summer encephalitis] | A84.0 | 063.0 |
| Central European tick-borne encephalitis | A84.1 | 063.2 |
| Other tick-borne viral encephalitis  Including Louping ill | A84.8 | 063.8/063.1 |
| Tick-borne viral encephalitis, unspecified | A84.9 | 063.9 |
| Other viral encephalitis, not elsewhere classified | A85 | 064 |
| Enteroviral encephalitis | A85.0 | 048 |
| Adenoviral encephalitis | A85.1 | 048 |
| Adenoviral encephalitis, myelitis and encephalomyelitis | A85.1 | 323.01 |
| Arthropod-borne viral encephalitis, unspecified | A85.2 | 064 |
| Other specified viral encephalitis | A85.8 | 323.8 |
| Unspecified viral encephalitis | A86 | 049.9 |
| Venezuelan equine encephalitis | A92.2 | 066.2 |
| West Nile virus infection with encephalitis | A92.31 | 066.41 |
| Herpes viral encephalitis | B00.4 | 054.3 |
| Varicella encephalitis, myelitis and encephalomyelitis | B01.1 | 052.0 |
| Postchickenpox encephalitis, myelitis and encephalomyelitis | B01.1 | 052.0 |
| Varicella encephalitis and encephalomyelitis | B01.11 | 052.0 |
| Zoster encephalitis | B02.0 | 053.0 |
| Measles complicated by encephalitis | B05.0 | 055.0 |
| Encephalitis, myelitis and encephalomyelitis in measles | B05.0 | 055.0 |
| Rubella encephalitis | B06.01 | 056.01 |
| Rubella encephalitis, myelitis and encephalomyelitis | B06.01 | 056.01 |
| Other human herpesvirus encephalitis | B10.0 | 058.29 |
| Human herpesvirus 6 encephalitis | B10.01 | 058.29 |
| Other human herpesvirus encephalitis | B10.09 | 058.29 |
| Cytomegaloviral encephalitis, myelitis and encephalomyelitis | B25.8 | 078.5 |
| Mumps encephalitis, myelitis and encephalomyelitis | B26.2 | 072.0 |
| Mumps encephalitis | B26.2 | 072.0 |
| Blastomycotic meningoencephalitis | B40.81 | 116.0 |
| Toxoplasma meningoencephalitis | B58.2 | 130.0 |
| Meningoencephalitis due to free-living amebae | B60.2 | 136.29 |
| Eosinophilic meningoencephalitis | B83.2 | 323.8 |
| Encephalitis, myelitis and encephalomyelitis | G04 | 323.9 |
| Acute disseminated encephalitis and encephalomyelitis (ADEM) | G04.0 | 323.6 |
| ADEM, unspecified | G04.00 | 323.6 |
| Postinfectious ADEM | G04.01 | 323.61 |
| Postimmunization ADEM | G04.02 | 323.5 |
| Bacterial meningoencephalitis and meningomyelitis, not elsewhere classified | G04.2 | 323.81 |
| Other encephalitis, myelitis and encephalomyelitis | G04.8 | 323.81 |
| Other encephalitis and encephalomyelitis | G04.81 | 323.81/049.8 |
| Encephalitis, myelitis and encephalomyelitis, unspecified | G04.9 | 323.9 |
| Encephalitis and encephalomyelitis, unspecified | G04.90 | 323.9 |
| Encephalitis, myelitis and encephalomyelitis in diseases classified elsewhere | G05 | 323.0/323.1/323.2 |
| Encephalitis and encephalomyelitis in diseases classified elsewhere | G05.3 | 323.4/323.41 |
| Toxic encephalitis | G92.8 | 323.7 |
| Benign myalgic encephalomyelitis | G93.3 | 323.8 |
| Encephalitis, myelitis and encephalomyelitis in systemic lupus erythematosus | M32.19 | 710.0 + 323.80 |
