# Supplementary material for: Repeat Lumbar Puncture in Adults With Encephalitis: Patient Characteristics, Cerebrospinal Fluid Dynamics, and Diagnostic Yield
Source: Open Forum Infect Dis. 2026 Sep 5;13(9):ofag574. doi: 10.1093/ofid/ofag574 (PMC13579470; doi:10.1093/ofid/ofag574)
Supplement: Supplementary file 1 [file ofag574_supplementary_data.zip › Supplementary Table 2.docx]

**Supplementary Table 2:** Characteristics of patients who received corticosteroids before versus after repeat lumbar puncture

| **Variable** | **Steroids before repeat LP**  **(n=39)** | **No steroids before repeat LP**  **(n=88)** | **P-value**^a^ |
| --- | --- | --- | --- |
| **Demographics** | | | |
| Male | 23/39 (59.0) | 44/88 (50.0) | 0.441 |
| Female | 16/39 (41.0) | 44/88 (50.0) |  |
| Age^b^, median [IQR], years | 40.5 [31.5–50.8] | 49.0 [30.0–64.0] | 0.009* |
| **Encephalitis Subtype** | | | |
| Autoimmune | 9/39 (23.1) | 31/88 (35.2) | 0.216 |
| Infectious | 10/39 (25.6) | 20/88 (22.7) | 0.821 |
| Unknown | 20/39 (51.3) | 37/88 (42.0) | 0.342 |
| **Comorbidities** |  |  |  |
| Immunocompromised^d^ | 11/39 (28.2) | 21/88 (23.9) | 0.660 |
| CCI, median [IQR] | 0.0 [0–2] | 1.0 [0–4] | 0.412 |
| CCI ≥3 | 9/39 (23.1) | 28/88 (31.8) | 0.398 |
| **Clinical Presentation** | | | |
| Acute onset | 18/38 (47.4) | 29/87 (33.3) | 0.162 |
| Fever | 21/39 (53.8) | 43/82 (52.4) | 0.520 |
| Seizures | 13/39 (33.3) | 51/86 (59.3) | 0.012* |
| Status epilepticus | 4/39 (10.3) | 18/88 (20.5) | 0.208 |
| Refractory status epilepticus | 0/36 (0.0) | 8/83 (9.6) | 0.104 |
| Headache | 19/36 (52.8) | 41/78 (52.6) | 0.572 |
| Altered mental status | 38/39 (97.4) | 77/88 (87.5) | 0.104 |
| Focal neurological signs^e^ | 16/39 (41.0) | 39/88 (44.3) | 0.846 |
| Psychiatric symptoms on admission | 22/38 (57.9) | 50/88 (56.8) | 0.535 |
| Memory deficits | 19 (48.7) | 30/87 (34.5) | 0.167 |
| Cerebral edema | 2/37 (5.4) | 14/87 (16.1) | 0.145 |
| **Laboratory Findings** | | | |
| CSF WBC change on repeat LP |  |  |  |
| Increase in CSF WBC count | 10/37 (27.0) | 39/85 (45.9) | 0.070 |
| Decrease or no change in CSF WBC count | 27/37 (73.0) | 47/85 (54.1) | 0.070 |
| **Hospitalization Outcomes** | | | |
| Length of stay, median [IQR], days^c^ | 18.5 [13.0–33.0] | 29.0 [13.0–45.0] | 0.019* |
| ICU admission | 20/39 (51.3) | 52/88 (59.1) | 0.442 |
| **Glasgow Outcome Scale (GOS)** | | | |
| Death (GOS 1) | 2 /39 (5.1) | 7/88 (8.0) | 0.721 |
| Persistent vegetative state (GOS 2) | 2/39 (5.1) | 10/88 (11.4) | 0.341 |
| Severe disability (GOS 3) | 18 /39 (46.2) | 49/88 (55.7) | 0.342 |
| Moderate disability (GOS 4) | 7/39 (17.9) | 6/88 (6.8) | 0.108 |
| Good recovery (GOS 5) | 10 /39 (25.6) | 7/88 (8.0) | 0.011* |

Data are presented as n/N (%) unless otherwise indicated. “*” indicates statistical significance or *P* < .05.**Abbreviations**: LP, lumbar puncture (LP1, first lumbar puncture during acute admission); CCI, Charlson comorbidity index; CSF, cerebrospinal fluid; EEG, electroencephalography; GCS, Glasgow Coma Scale; ICU, intensive care unit; IQR, interquartile range; LOS, length of stay; MRI, magnetic resonance imaging; GOS, Glasgow Outcome Scale; WBC, white blood cell count.

^a^*P* value comparing the patients who received steroids before repeat LP with patients who did not receive steroids

^b^Age range, 18–92 y.

^c^Length of stay at the hospital.

^d^Immunocompromised is defined as HIV, recent chemotherapy (<1 m), solid-organ or bone marrow transplantation, receiving ≥20 mg of prednisone or equivalent for >1 m, or congenital immunodeficiency.

^e^Self-reported symptoms suggestive of focal neurological manifestations such as acute-onset cranial nerve abnormalities or acute defects in sensorimotor abilities, including aphasia
